# CD77 levels over enzyme replacement treatment in Fabry Disease Family (V269M)

**DOI:** 10.1590/2175-8239-JBN-3910

**Published:** 2018-06-04

**Authors:** Ester Miranda Pereira, Adalberto Socorro da Silva, Raimundo Nonato da Silva, José Tiburcio Monte, Fernando F. do Nascimento, Jackeline L. M. Sousa, Henrique César Saraiva de Arêa Leão Costa, Herton Luiz Alves Sales, Anatalia Labilloy, Semiramis Jamil Hadad do Monte

**Affiliations:** 1 Universidade Federal do Piauí, Teresina, PI, Brasil.

**Keywords:** Fabry Disease, Enzyme Replacement Therapy, Phagocytes, Doença de Fabry, Terapia de Reposição de Enzimas, Fagócitos

## Abstract

**Introduction:**

Fabry disease (FD) is a disorder caused by mutations in the gene encoding for lysosomal enzyme α-galactosidase A (α-GAL). Reduced α-GAL activity leads to progressive accumulation of globotriaosylceramide (Gb3), also known as CD77. The recent report of increased expression of CD77 in blood cells of patients with FD indicated that this molecule can be used as a potential marker for monitoring enzyme replacement therapy (ERT).

**Objective:**

The purpose of this study was to evaluate the CD77 levels throughout ERT in FD patients (V269M mutation).

**Methods:**

We evaluated the fluctuations in PBMC (peripheral blood mononuclear cell) membrane CD77 expression in FD patients undergoing ERT and correlated these levels with those observed in different cell types.

**Results:**

A greater CD77 expression was found in phagocytes of patients compared to controls at baseline. Interestingly, the variability in CD77 levels is larger in patients at baseline (340 - 1619 MIF) and after 12 months of ERT (240 - 530 MIF) compared with the control group (131 - 331 MFI). Furthermore, by analyzing the levels of CD77 in phagocytes from patients throughout ERT, we found a constant decrease in CD77 levels.

**Conclusion:**

The increased CD77 levels in the phagocytes of Fabry carriers together with the decrease in CD77 levels throughout ERT suggest that measuring CD77 levels in phagocytes is a promising tool for monitoring the response to ERT in FD.

## RESUMO

**Introdução:**

A doença de Fabry (DF) é um distúrbio causado por mutações no gene que codifica a enzima lisossômica α-galactosidase A (α-GAL). A redução da atividade de α-GAL leva ao acúmulo progressivo de globotriaosilceramida (Gb3), também conhecida como CD77. O recente relato de aumento da expressão de CD77 em células sanguíneas de pacientes com DF indicou que essa molécula pode ser utilizada como um potencial marcador para o monitoramento da terapia de reposição enzimática (TRE).

**Objetivo:**

O objetivo deste estudo foi avaliar os níveis de CD77 ao longo da TRE em pacientes com DF (mutação V269M).

**Métodos:**

Foram avaliadas as flutuações na expressão de CD77 nas membranas das CMSP (células mononucleares do sangue periférico) em pacientes com DF submetidos à TRE e correlacionados com aqueles observados em diferentes tipos de células. Resultados: Uma maior expressão de CD77 foi encontrada em fagócitos de pacientes em comparação aos controles no início do estudo. Curiosamente, a variabilidade nos níveis de CD77 é maior em pacientes no início do estudo (340 - 1619 MIF) e após 12 meses de TRE (240 - 530 MIF) em comparação com o grupo controle (131 - 331 MFI). Além disso, analisando os níveis de CD77 em fagócitos de pacientes ao longo da TRE, encontramos uma diminuição constante nos níveis de CD77.

**Conclusão:**

O aumento nos níveis de CD77 nos fagócitos de portadores de Fabry, juntamente com a diminuição nos níveis de CD77 ao longo da TRE, sugerem que medir os níveis de CD77 nos fagócitos é uma ferramenta promissora para monitorar a resposta à TRE na DF.

## INTRODUCTION

The GLA gene (300644) located at position Xq22.1 on the human X chromosome constitutively encodes the lipase alpha galactosidase A enzyme (EC 3.2.1.22, a-GAL) under normal conditions.^1^ Different mutations in this gene may result in a total lack or decreased activity of a-GAL, which results in Fabry Disease (FD; OMIM #301500).^2^ This is a rare syndrome in which the average time between the appearance of its first symptoms (acroparesthesia, hypo- or anhidrosis, angiokeratoma, abdominal pain, and Fabry-associated pain)^1^^,^ 3 ^-^ 6 and the clinical diagnosis is approximately 15 years in men and 40 years in women.^7^ The biological manifestations observed in FD come from the non-degradation of glycosphingolipids by a-GAL within the lysosomes, which results in the progressive accumulation of compounds, such as globotriaosylceramide (Gb3).^8^

Enzyme replacement therapy (ERT) carried out in an endovenous manner with specific protocols has brought hope for the treatment of FD; currently, it is the only therapy for the disease accepted worldwide. However, despite its success, effectively monitoring ERT is still a challenge, as it is based on Gb3 and lyso-Gb3 levels in plasma and urine, clinical improvements in renal function, and reduction of left ventricular hypertrophy. However, the use of these biochemical parameters as trustworthy tools for monitoring the effectiveness of ERT in FD was recently questioned by studies showing that (i) there is no correlation between FD severity and Gb3 and lyso-Gb3 levels, and (ii) FD patients may display normal Gb3 and lyso-Gb3 levels in body fluids.^9^^-^ 13

The finding that blood cells from FD individuals express membrane-bound CD77 (Gb3 bonded to the membrane) at fourfold higher levels than healthy controls^14^ together with the finding that HK2 cells (overexpressing CD77 by silencing the a-GAL gene) decrease CD77 expression after three days of treatment with ERT^14^ have encouraged studies to investigate the variations in CD77 levels throughout ERT in FD patients. Therefore, we aimed to study the CD77 levels in PBMC (peripheral blood mononuclear cell) membrane in FD patients undergoing ERT; the levels were correlated with those observed in different cell types in subgroups of age and gender.

## METHODS

### POPULATION

This study was approved by the Ethical Review Board of the Federal University of Piauí (CEP-0160.0.045.000-10) and carried out with 25 patients diagnosed with FD (confirmed by clinical and molecular tests). All the patients come from a large family carrying the V269M mutation in the gene that encodes a-GAL A, which was recently discovered by our group in southern Piauí, Brazil. The patients were categorized into the following groups: group 1 (GP1, n = 17), including individuals with classical disease phenotypes who were under ERT (algasidase alpha, 0.2 mg/Kg, fortnightly), and group 2 (GP2, n = 8), including individuals who did not undergo ERT. One hundred and one healthy volunteers were studied as a control group. The subjects signed a Free and Informed Consent Form and had their biological samples (peripheral blood) processed at the Laboratory of Immunogenetics and Molecular Biology at the Federal University of Piauí.

### BASELINE AND FOLLOW-UP OF BIOCHEMICAL AND CLINICAL EVALUATION

Baseline data and specific findings and symptoms relevant to FD were recorded systematically in a database and used for further analyses. The patients' medical histories indicated the presence of angiokeratoma, acroparesthesia, pain episodes, hypohidrosis, hypertension, cornea verticillata, and proteinuria. Left ventricular hypertrophy (LVH) was defined as an interventricular septal and/or posterior wall thickness in end-diastole ≥ 13 mm detected by echocardiography and renal function was quantified by the estimated glomerular filtration rate (eGFR) using the Chronic Kidney Disease-Epidemiology Collaboration equation (CKD-EPI).^15^ Renal impairment was defined as eGFR < 90 mL/min/1.73 m^2^ according to "Kidney Disease: Improving Global Outcomes" (KDIGO)^16^ and the European FD^17^ guidelines. Albumin and creatinine concentrations in urine samples were determined using a LabTestAlbumin kit and a LabTestCreatinine Kit (Minas Gerais, Brazil), respectively, according to the manufacturer's protocol. Urine albumin and creatinine ratio (ACR) was calculated from the concentrations of urine albumin and urine creatinine, and the values were reported in mg/g. 

### FLOW CYTOMETRY

Fresh peripheral blood cells (2 x 10^5^) were incubated in 2.5 mL of PBS for 15 min at 4°C in the dark with the relevant antibodies. Fluorescently labeled monoclonal antibodies (Becton-Dickinson, Mountain View, CA, USA) directed at the following molecules were used: membrane pan leukocyte marker CD45 (allophycocyanin (APC)-conjugated), cytoplasmic CD77 (fluorescein isothiocyanate (FITC)-conjugated), and membrane CD11b (phycoerythrin (PE)-conjugated. Intracellular labeling of CD77 was achieved by hole-punching the cells using BD FACS Lyse solution accordingly (BD Bioscience, USA). Peripheral blood cells were examined using a FACSCanto II flow cytometer and were gated based on forward scatter (FSC) and side scatter (SSC) measures. The results were analyzed using the Facs Diva software (Becton-Dickinson) and reported as the median fluorescence intensity (MFI).

### STATISTICAL ANALYSIS

The Mann-Whitney non-parametric test was used both to compare the expression differences (MFI) in CD77 between control and patients and to compare the expression differences in CD77 in the patients cells as a function of age (< 50 *vs*. ≥ 50 y.o.). The Wilcoxon test was used to compare the changes in the biochemical parameters LVMI, ACR, and eGFR before and after ERT.

## RESULTS

**Figure 1:**
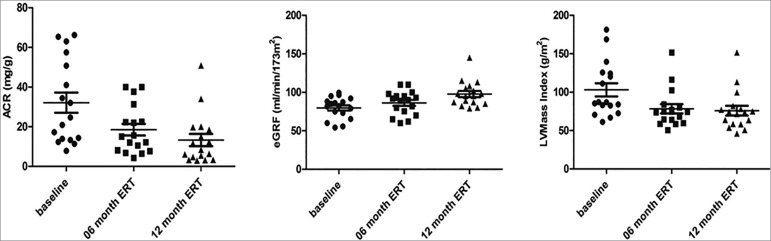
Follow up of clinical and laboratory parameters in FD patients (V269M) over ERT. A) ACR in baseline, 6, and 12 months of ERT. B) eGFR in baseline, 06, and 12 months after ERT. LVMI decreased significantly from baseline to 06 and 12 months of ERT. FD: Fabry disease; ACR: Albumin creatinine ratio; LVMI: left ventricular mass index; eGFR: estimated glomerular filtration rate.; ERT: Enzyme Replacement Therapy.

From 2005 to 2010, our group diagnosed 64 people with FD (V269M) from the same family in southern Piauí, Brazil. Among these, only those who were under follow-up every 6 months were included in the study. Demographic, clinical, and laboratory data for the GP1 and GP2 patients are found in Table 1. The patients in the GP1 group ranged in age from 13 to 75 years, and the follow-up time ranged from 06 to 21 months. In the GP2 group, the ages ranged from 8 to 50 years. No patient had a history of ischemic cerebrovascular accident (CVA) and/or syncope. Follow-up evaluations in the GP1 patients showed a significant decrease in ACR after 6 (*p* = 0.0010) and 12 (*p* = 0.0016) months of treatment (Figure 1-A) and increase in the eGFR parameter after 6 (*p* < 0.0001) and 12 (*p* < 0.0001) months (Figure 1-B). We found decreases in the LVMass Index in the follow-up treatments after 6 and 12 months (*p* = 0.004 and *p* = 0.003, respectively) (Figure 1-C).

**Table 1:** Demographic, clinical, and laboratory data for the GP1 and GP2 Fabry disease patients (V269M)

Demographic data	GPI	GP2
Age (years)	40.4 ± 4.5	24.5 ± 4.4
Male (n)	6	2
Female (n)	11	6
Clinical and Laboratory data		
Angiokeratoma (%)	0	0
ECG abnormalities (%)	33.3	0
LVH (%)	13.3	12.5
Cornea verticilata (%)	33.3	50
Acroparestesia (%)	100	37.5
Hypertension (%)	52.9	12.5
Radiographic Lesions in MRI (%)	41.2	37.5
eGFR < 90 mL/min/1.73 m^2^ (%)	52.9	0

ECG: electrocardiogram; LVH: left ventricular hypertrophy ; eGFR: estimated glomerular filtration rate; MRI: magnetic resonance imaging.

**Figure 2:**
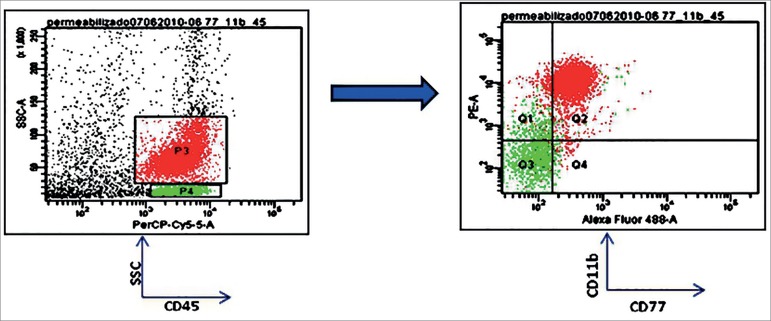
Phagocytes cells have higher CD77 levels than lymphocytes. Red population represent monocyte/neutrophil cells. Green population represent lymphocytes.

**Figure 3:**
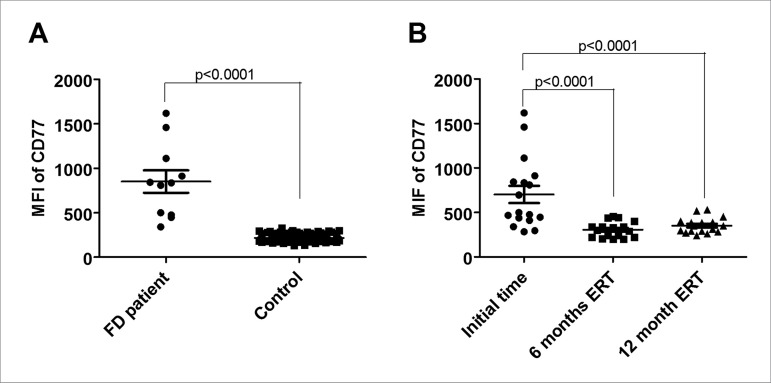
CD77 levels in FD patients' phagocytes over the ERT. A) MFI of CD77 is increased in FD patients' phagocytes compared to controls. B) CD77 levels decreased after first six months compared with the baseline (MFI = 702.4 ± 396.4 *vs*. MFI = 306.6 ± 89.43; *p* < 0.0001) followed by a stabilization in the CD77 levels. FD: Fabry disease; MFI: median fluorescence intensity; ERT: Enzyme Replacement Therapy.

**Figure 4:**
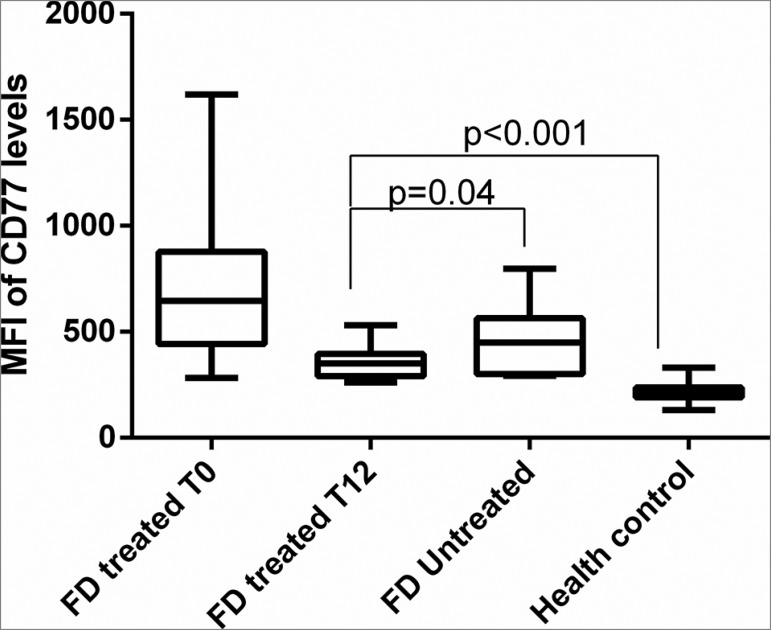
CD77 levels in GP1 and GP2 FD patients. After one year of ERT, CD77 levels were lower in the GP1 patients (MFI = 356.0 ± 20.21) than in the GP2 patients (MFI = 462.3 ± 62; *p* = 0.04) but were still higher than in healthy controls (MFI = 216.9 ± 3.235; *p* < 0.001). FD: Fabry disease; MFI: median fluorescence intensity; ERT: Enzyme Replacement Therapy.

To determine the differential locations of CD77 in different populations of leukocytes, we assessed the MFI of these molecules in the lymphocyte and monocyte/neutrophil (phagocyte) populations. We observed high values in phagocytes but not in lymphocytes in both patient and control groups (Figure 2). We observed a significant increase in CD77 density in phagocytes of GP1 compared to controls (MFI = 702.4 ± 396.4 *vs*. MFI = 217 ± 37.6; *p* < 0.0001) (Figure 3-A). We then investigated the impact of ERT on CD77 levels in the phagocytic population over a year of ERT treatment. Our analysis revealed a two-fold reduction in CD77 levels during the first six months compared with baseline (MFI = 702.4 ± 396.4 *vs*. MFI = 306.6 ± 89.43; *p* < 0.0001) (Figure 3-B), followed by a stabilization in the CD77 levels in these patients. Afterwards, we compared the CD77 levels from GP1 at T0 and T12 and compared with GP2 and healthy controls. After one year of ERT, CD77 levels were lower in the GP1 patients (MFI = 356.0 ± 20.21) than in the GP2 patients (MFI = 462.3 ± 62; *p* = 0.04) but were still higher than those of the healthy controls (MFI = 216.9 ± 3.235; *p* < 0.001) (Figure 4).

**Figure 5:**
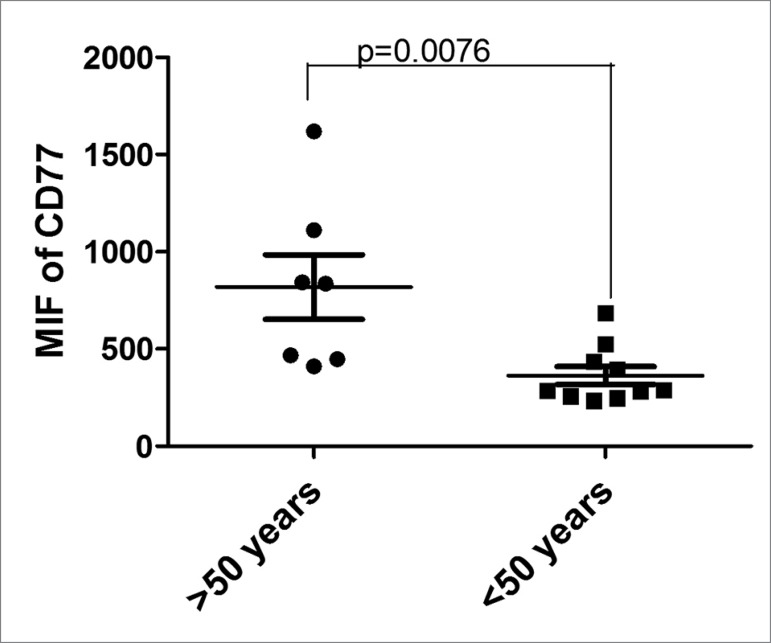
CD77 levels in phagocytes cells of FD patients older than and younger than 50 years. GP1 patients aged ≥ 50 had higher CD77 levels compared to patients < 50 years (MIF = 819 ± 165.8 *vs*. MFI = 363 ± 46.3; *p* = 0.0076). FD: Fabry disease; MFI: median fluorescence intensity.

To determine the effect of age, GP1 patients were stratified into the subgroups < 50 and ≥ 50 years old. The analysis revealed higher CD77 levels in phagocytes of patients aged ≥ 50 (MIF = 819 ± 165.8 *vs*. MFI = 363 ± 46.3; *p* = 0.0076) (Figure 5).

To assess the relationship between CD77 levels in phagocytic cells and clinical seriousness, we correlated CD77 levels with the variables AUC, eGRF, and LVMI; no significant correlation was found.

## DISCUSSION

We studied the levels of CD77 in leukocytes of FD patients undergoing or not ERT and in healthy controls. We found a different expression pattern for this molecule in different leukocyte populations in patients and controls. CD77 is preferably expressed in phagocytic cells regarded as lymphocytes. Reports in the literature show that high levels of CD77 at the germinal center quickly induce apoptosis of B cells^18^ and that there is a high CD77 expression in patients with Burkitt lymphomas.^19^ These findings suggest that CD77 participates in the regulation of the B lineage differentiation at the germinal center. The role of CD77 in other hematopoietic cells has not yet been elucidated. Nevertheless, CD77 is known to be a receptor for *Escherichia coli* verotoxin in epithelial, intestinal, and renal cells^20^ and is significantly increased in colorectal carcinomas and their metastases.^21^^,^ 22

The deficiency in a-GAL in FD leads to the progressive accumulation of Gb3 within cells. Gb3 is identical to the CD77 membrane antigen. Therefore, an increase in CD77 levels is expected in FD. Actually, we observed a greater CD77 expression in phagocytes of patients compared to controls at baseline. Interestingly, the variability in CD77 levels was larger in patients at time 0 (340 - 1619 MFI) and after 12 months of ERT (240 - 530 MFI) compared with the control group (131 - 331 MFI). Furthermore, we found a constant decrease in CD77 levels in phagocytes from GP1 patients throughout ERT, reaching levels as low as those in GP2 but still higher than in controls. Similar results were attained in a study by Thomaidis and collaborators (2009). The authors developed the first cell model to study FD using gene silencing techniques. They measured CD77 levels by flow cytometry and Gb3 levels by mass spectrometry in a-GAL-deficient cells and found Gb3 accumulation as well as CD77 overexpression upon comparison with non-silenced cells. To confirm these findings, they measured CD77 levels in polymorphonuclear cells from FD carriers and compared with the levels in healthy individuals, finding higher levels in patient cells (fourfold). To determine whether CD77 expression was related to the reduction in a-GAL activity, they compared CD77 levels in silenced cells treated with and without ERT and showed that the treated cells displayed lower levels compared with the non-treated cells.^14^

There are currently two markers for diagnosing and monitoring ERT, Gb3, and Lyso-Gb3. In classical FD, Gb3 can be reliably measured in plasma and urine using mass spectrometry.^23^ However, there are large variations in the concentrations detected. It seems that some patients with mild mutations in the gene that encodes α-GAL, which are not associated with an absolute enzyme deficiency, may display normal Gb3 levels in body fluids. Aerts *et al*. (2008) have recently suggested that the deacetylated form of Gb3 (lyso-Gb3) is also a marker for clinical management of FD. Although lyso-Gb3 circulation is significantly increased in male patients with FD, it was only partially responsive to ERT with recombinant a-GAL A, and there was no relationship between plasma lyso-Gb3 levels and age or clinical evolution.^9^

The increased CD77 levels in the phagocytes of Fabry carriers together with its decrease throughout ERT suggest that measuring CD77 levels in phagocytes is a promising tool for monitoring the response to ERT in FD. The levels are higher in patients than in healthy controls even after ERT, distinguishing between carriers and non-carriers of the disease. Additionally, because this measurement is accomplished by flow cytometry, which is a simple, fast, and universal technique, this tool may be incorporated into laboratorial routines for FD. Although the CD77 levels in the phagocytes of FD carriers are not correlated with clinical variables, it was possible to observe clinical improvement in these patients as the levels of CD77 decreased. The absence of this correlation may be due to a different cell turnover in blood compared to cardiac and renal cells.

Even though our study was carried out in a single FD carrier family and CD77 levels did not correlate with clinical variables, our study demonstrated the differential location of CD77 in blood cells and monitored CD77 levels in phagocytes throughout ERT in FD patients. Our results also provide new perspectives for understanding the importance of CD77 in FD physiopathology and treatment.
